# Can Intraoperative Low-Dose *R*,*S*-Ketamine Prevent Depressive Symptoms After Surgery? The First Meta-Analysis of Clinical Trials

**DOI:** 10.3389/fphar.2020.586104

**Published:** 2020-10-19

**Authors:** Liwei Pang, Meiying Cui, Wanling Dai, Jing Kong, Hongzhi Chen, Shuodong Wu

**Affiliations:** ^1^ Department of General Surgery, Shengjing Hospital of China Medical University, Shenyang, China; ^2^ Department of Anesthesiology, Shengjing Hospital, China Medical University, Shenyang, China; ^3^ Innovation Institute of China Medical University, Shenyang, China

**Keywords:** postoperative depression, (*R*,*S*)-ketamine, meta-analysis, surgery, antidepressant

## Abstract

**Background:** Postoperative depression is a common complication after surgery that profoundly affects recovery and prognosis. New research indicates that (*R*,*S*)-ketamine is a potent antidepressant that exerts a rapid and sustained antidepressive effect. However, there is no consensus on whether intraoperative low-dose (*R*,*S*)-ketamine prevents postoperative depression.

**Objectives:** This study aimed to investigate the safety, feasibility, and short-term complications of intraoperative low-dose (*R*,*S*)-ketamine in preventing postoperative depressive symptoms.

**Methods:** The Web of Science, Cochrane, PubMed, and CNKI databases were systematically searched (last search February 28, 2020) to identify studies involving ketamine. Sensitivity and metaregression analyses were performed to identify potential confounders. The meta-analysis was performed using Review Manager 5.3.

**Results:** A total of 13 studies (seven in Chinese and six in English) representing 1,148 cases of patients who were treated with (*R*,*S*)-ketamine and 874 cases of patients who received other treatments were included in the meta-analysis. Anesthesia duration and blood loss did not significantly differ between the two groups, demonstrating that (*R*,*S*)-ketamine was safe (odds ratio,OR: 0.27; 95% CI: −1.14 to 1.68; P = 0.71) for prophylactic treatment of postoperative depression. Blood loss (OR: −1.83; 95% CI: −8.34 to 4.68; P = 0.58), the number of postoperative depressive patients (95% CI: 0.8–1.07; P = 0.08; (*R*,*S*)-ketamine: control = 12.9%:15.8%), and postoperative complications (OR: 0.83, 95% CI: 0.44–1.58; P = 0.57; (*R*,*S*)-ketamine: control = 19.3%:19.3%) were all similar across groups. Intra-operative low-dose (*R*,*S*)-ketamine reduced extubation time (OR: −2.84; 95% CI: −5.48 to −0.21; P = 0.03).

**Conclusions:** The prophylactic anti-depressant effect of (*R*,*S*)-ketamine did not significantly differ between the (*R*,*S*)-ketamine and control groups in patients undergoing general or spinal anesthesia. However, (*R*,*S*)-ketamine use led to a higher incidence of adverse reactions in patients under 40 years of age who underwent a Cesarean section under spinal anesthesia.

## Introduction

Postoperative depression is a perioperative psychological complication that severely affects patient recovery and quality of life (Kim et al., 2016; Visser et al., 2019). In extreme cases, it may lead to suicidal behavior. Postoperative depression can be seen in various surgical operations including thyroidectomy (Choi et al., 2019), mastectomy (Zhang et al., 2018), spinal surgery (Wilson et al., 2017), and cesarean section (Lurie, 2016). The mechanism underlying postoperative depression remains elusive, but interaction between multiple systems is certain. We postulate that the mechanism of postoperative depression is similar to that of major depressive disorder, which encompasses metabolic disorders of the serotonin system (Cervenka et al., 2017), glutamatergic (Murrough et al., 2017) and GABAergic (Duman et al., 2019) neurotransmission signaling dysfunction, abnormal brain connectivity (Clemm von Hohenberg et al., 2018), stress-induced neuroinflammation (Setiawan et al., 2015), and synaptoplastic dysfunction (Price and Duman, 2020). Complex factors induce postoperative depression, including the stress of the surgery itself, anesthetic drug neurotoxicity, postoperative pain, and mood disorders caused by a poor recovery after surgery. Postoperative depression cannot always be predicted based on previous medical and psychological history (Lenze et al., 2007). Approximately 20% of surgical patients suffer from perioperative depressive symptoms that can affect recovery and prognosis (Urban-Baeza et al., 2015). The incidence of postoperative depression may even reach 30% in heart surgeries such as coronary artery bypass grafting (Pietrzyk et al., 2014). Thus, effective methods to prevent postoperative depression are needed.

The intraoperative application of intravenous (*R*,*S*)-ketamine is an interesting potential intervention for postoperative depression because new research shows that (*R*,*S*)-ketamine is a potent antidepressant that exerts a rapid and sustained antidepressive effect and eliminates suicidal ideation at a subanesthetic dose (0.5 mg/kg). However, the preventive effect of (*R*,*S*)-ketamine on postoperative depression is uncertain because the intervention is distinct from that of (*R*,*S*)-ketamine in the treatment of treatment-resistant depression. Classic (*R*,*S*)-ketamine is a racemic mixture of R-ketamine and S-ketamine. In a mouse model, R-ketamine had a more potent and sustained antidepressant effect than S-ketamine (Zhang et al., 2014; Yang et al., 2015). An open-label pilot trial showed that Montgomery–Åsberg Depression Rating Scale scores dropped significantly without any signs of dissociation in patients with treatment-resistant depression following intravenous (*R*,*S*)-ketamine administration (0.5 mg/kg). This result indicates that R-ketamine exerts a rapid and sustained antidepressant effect in humans, consistent with the results seen in animal models (Leal et al., 2020). (*R*,*S*)-Ketamine can be administered intravenously and orally (Rosenblat et al., 2019), and *S*-Ketamine can be *via* a nasal spray (Pal, 2019; Slomski, 2019). The effects of (*R*,*S*)-ketamine are most rapidly seen when it is administered intravenously (Berman et al., 2000). The exact mechanism of the antidepressant effect of (*R*,*S*)-ketamine remains unclear. However, current hypotheses include increased central nervous expression of brain-derived neurotrophic factor (BDNF), activation of mammalian target of rapamycin signaling, increased expression of synaptic protein, promoted neurotransmission or synaptogenesis *via* alpha-amino-3-hydroxy-5-methyl-4-isoxazolpropionic acid receptor activation (Cavalleri et al., 2018), and inhibited neuronal burst firing in the lateral habenula (Yang et al., 2018). In addition to its antidepressant effect, (*R*,*S*)-ketamine also exerts a prophylactic effect on depression (Mastrodonato et al., 2020). Under inflammatory stress, (*R*,*S*)-ketamine triggers neuroprotective activity and has a prophylactic effect on stress-induced depression-like behavior in mice (Brachman et al., 2016). The prophylactic effect of (*R*,*S*)-ketamine is associated with neuroplasticity in the CA3 region of the hippocampus (Mastrodonato et al., 2018; Krzystyniak et al., 2019). In recent years, there have been several clinical studies on the prophylactic effect of (*R*,*S*)-ketamine on postoperative depressive symptoms, but none of these studies were systematic reviews or meta-analyses (Sall et al., 2019; Highland et al., 2020). The aim of this study was to collect and analyze previous studies to provide a more systematic evaluation of the prophylactic effect of (*R*,*S*)-ketamine on postoperative depression.

## Methods

This study was designed according to the Preferred Reporting Items for Systematic Reviews and Meta-Analyses (PRISMA) guidelines. Medical databases were searched for articles using the following terms: depression, ketamine, surgery, and operative. This search strategy was designed and executed by an experienced information specialist and reviewed by two writers (LP and MC).

### Inclusion Criteria

Inclusion criteria were as follows:

1. Study design: Clinical trials.
2. Interventions: Studies comparing ketamine and other factors.
3. Participants: Patients who underwent general anesthesia or spinal anesthesia.
4. Language: Without restriction to languages.
5. Type of article: Only studies published as full-text articles.
6. Studies included at least 10 patients.

### Exclusion Criteria

1. Noncomparable or nonhuman studies, review articles, editorials, letters, and case reports.
2. Articles not reporting the outcomes of interest.

### Literature Analysis

A detailed literature search was performed using depression, ketamine, surgery, and operative as keywords in the Web of Science (169), Cochrane (106), PubMed (230), and CNKI (351) online databases (last search date: February 28, 2019) without region, publication type, or language restrictions. The search strategy applied to PubMed is listed below (any keyword containing multiple forms, including its noun, adjective, or any other form could be replaced by *):

#1:depression*
#2:ketamine
#3:surgery*
#4:operative*
#5:(#3) OR (#4)
#6:(#1) AND (#2) AND (#5)

When similar reports describing the same population were published, the most recent or complete report was used. The research was conducted independently by Liwei Pang and Meiying Cui, and all authors subsequently compared their results. Article references were investigated manually, and any differences were resolved by consensus. This meta-analysis adhered to the guidelines outlined in the PRISMA statement.

### Data Extraction

The following data were extracted: author names, study design, number of patients, surgical method, anesthesia type, operation time, estimated blood loss, postoperative complications, depression score, visual analogue scale (VAS) score, and number of depressive patients.

### Quality Assessment and Statistical Analysis

Studies were rated based on the level of evidence provided according to the criteria by the Centre for Evidence-Based Medicine (Oxford, United Kingdom). Methodological quality was assessed using the modified Newcastle–Ottawa Scale (Table 1) and consisted of three factors: patient selection, study group comparability, and outcome assessment. A score of 0–9 was allocated to each study, and observational studies achieving a score of six or higher were considered high quality.

**TABLE 1:** Assessment of study quality based on the modified Newcastle–Ottawa Scale.

Author	Is the case definition adequate?	Representativeness of the cases	Selection of controls	Definition of controls	Comparability of cases and controls on the basis of the design or analysis	Ascertainment of exposure	Same method of ascertainment for cases and controls	Nonresponse rate	Total score
Akira	☆	☆	☆		☆☆	☆	☆	☆	8
Liu	☆	☆	☆	☆	☆	☆	☆	☆	8
Lv	☆	☆	☆	☆	☆	☆	☆	☆	8
Zhang	☆	☆	☆	☆	☆	☆	☆	☆	8
Min	☆	☆	☆	☆	☆☆	☆	☆	☆	9
Zhou	☆	☆	☆		☆	☆	☆	☆	7
Dai	☆	☆	☆	☆	☆	☆	☆	☆	8
Xu	☆	☆	☆		☆	☆	☆	☆	7
Yang	☆	☆	☆	☆	☆☆	☆	☆	☆	9
Mashour	☆	☆	☆	☆	☆☆	☆	☆	☆	9
Li	☆	☆	☆		☆	☆	☆	☆	7
Peirong	☆	☆	☆	☆	☆	☆	☆	☆	8
Wang	☆	☆	☆		☆	☆	☆	☆	7

Review Manager 5.3 (Cochrane Collaboration, Oxford, England) was used for all statistical analyses. Considering that patients were selected by different surgical teams and operated on at different centers, a random-effects model was chosen to assess heterogeneity. *I* ^*2*^ was used to assess heterogeneity, and values above 50% were considered substantial. Continuous variables were analyzed using the weighted mean difference.

## Results

**FIGURE 1:**
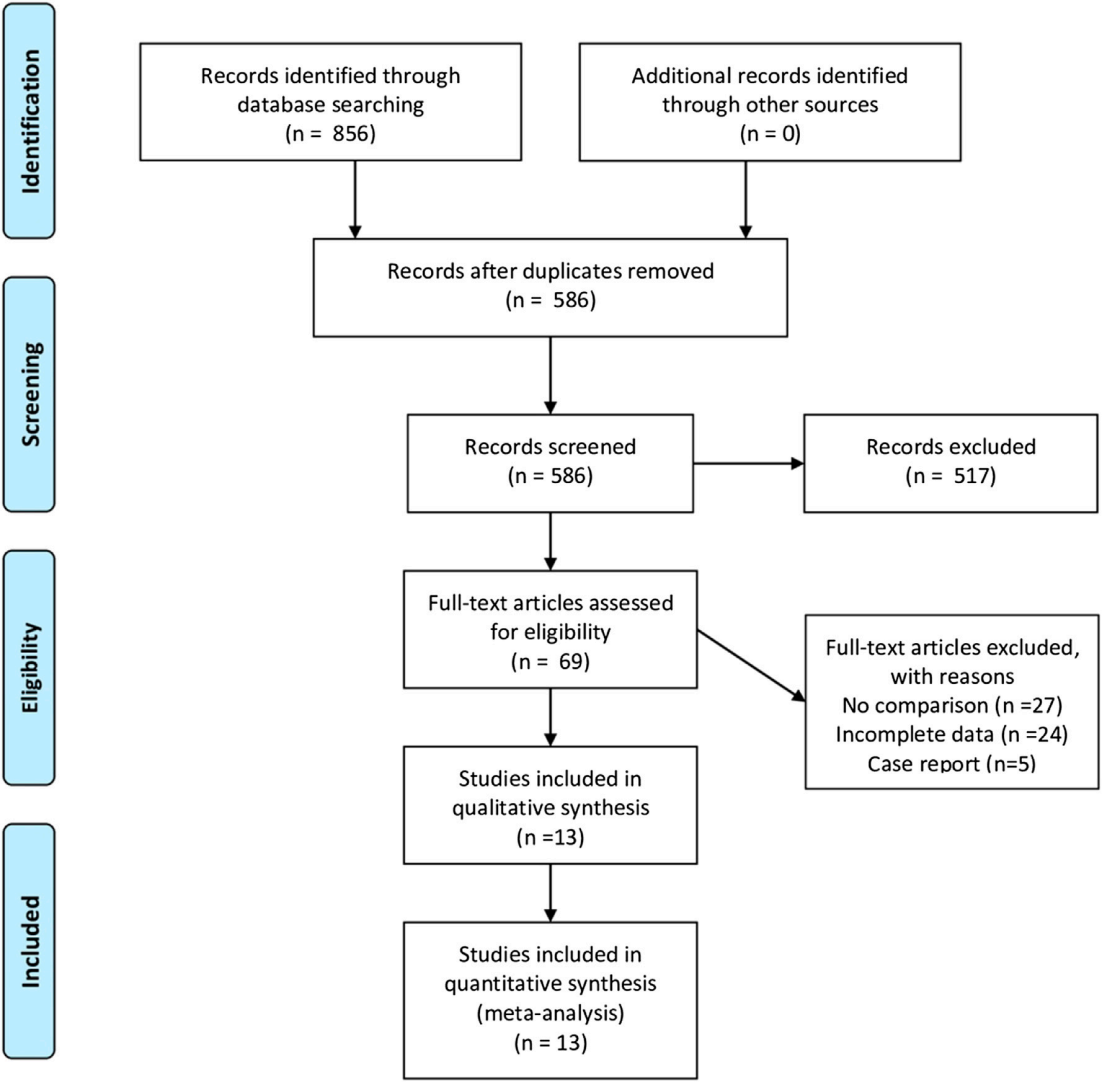
Preferred Reporting Items for Systematic Reviews and Meta-Analyses flow diagram.

A total of 856 studies that met the search criteria were initially found. No other eligible studies were found from other sources. After reading the titles and abstracts, 69 articles were included for a full-text evaluation. Twenty-four articles were excluded because the data were not impactful or the authors did not provide sufficient detail. Another 27 studies lacked a comparative analysis and were excluded. Besides, five were case reports. Finally, 13 studies (Kudoh et al., 2002; Liu and Zhang, 2013; L, 2015; Jiang et al., 2016; Zhang and Wang, 2016; Dai and Wang, 2017; Xu R. et al., 2017; Xu Y. et al., 2017; Zhou and Gan, 2017; Mashour et al., 2018; Li and Hou, 2019; Liu et al., 2020; Wang and Yang, 2020) (seven in Chinese and six in English) representing 1,148 cases of patients treated with ketamine and 874 cases of patients treated with other factors were included in the meta-analysis. Figure 1 illustrates the PRISMA flowchart of literature search strategies, and Table 2 describes the included articles. Because different articles had different scoring methods and definitions for depression, only data mentioned in the original articles were used to draw bar charts (Table 3).

**TABLE 2:** Summary of the data extracted from articles included in this meta-analysis.

Author	Year	Sample size (N)	Intervention (experimental:control)	Intervention (control)	Anesthesia	Time point of ketamine injection	Surgery	Age (years)	Gender (M:F)	Duration of anesthesia (min)	Blood loss (ml)
Akira	2002	35/35/25	1.0 mg/kg of ketamine; 1.5 mg/kg of propofol	2 µg/kg of fentanyl	General anesthesia	Induction	Orthopedic surgery	46.9 ± 8.8/48.2 ± 7.4/46.2 ± 10.3	—	135.6 ± 40.0/141.5 ± 43.7/122.8/46.1	95.1 ± 25.2/100.3 ± 31.8/93.6 ± 27.4
Liu	2013	40/40	0.5 mg/kg of ketamine	0.5 mg/kg of saline	Spinal epidural anesthesia	After delivery of the baby	Cesarean section	28.4 ± 6.4/29.0 ± 5.9	0:40/0:40	54.8 ± 10.8/53.2 ± 11.3	—
Lv	2015	47/47	0.25 mg/kg of ketamine	0.25 mg/kg of saline	Spinal epidural anesthesia	After delivery of the baby	Cesarean section	30.1 ± 2.1/29.4 ± 2.4	0:47/0:47	—	—
Zhang	2016	30/30	0.5 mg/kg of ketamine	0.5 mg/kg of saline	Spinal epidural anesthesia	After delivery of the baby	Cesarean section	27.87 ± 4.37/27.77 ± 4.14	0:30/0:30	39.53 ± 4.96/40.37 ± 6.20	—
Min	2016	60/60	0.5 mg/kg ketamine + 0.25 mg/kg/h continuous infusion for 30 min	0.5 mg/kg saline + 0.25 mg/kg/h	General anesthesia	Given at induction of anesthesia	Elective orthopedic surgery	43.38 ± 0.95/41.40 ± 0.16	34:26/33:27	126.58 ± 6.21/125.50 ± 7.90	405.00 ± 91.71/421.67 ± 88.74
Zhou	2017	63/63	0.5 mg/kg of ketamine	0.5 mg/kg of saline	Spinal epidural anesthesia	Continuous infusion for 30 min	Cesarean section	27.4 ± 5.3/28.3 ± 5.6	0:63/0:63	—	—
Dai	2017	30/30	0.5 mg/kg ketamine + 0.25 mg/kg/h continuous infusion for 30 min	0.5 mg/kg saline + 0.25 mg/kg/h	General anesthesia	After induction	Esophageal cancer	58 ± 6/61 ± 6	24:6/23:7	239 ± 54/239 ± 40	178 ± 94/210 ± 79
Xu	2017	25/25	0.5 mg/kg of ketamine	0.5 mg/kg of saline	General anesthesia	One hour after induction	Breast cancer	42.36 ± 7.28/43.27 ± 6.60	—	159.09 ± 42.30/157.73 ± 52.79	—
Yang	2017	162/163	0.25 mg/kg of ketamine	0.25 mg/kg of saline	Spinal epidural anesthesia	Within 5 min following clamping of the neonatal umbilical cord	Cesarean section	31 ± 4/32 ± 4	0:162/0:163	43.8 ± 14.4/44.0 ± 12.6	—
Mashour	2018	226/223/221	0.5 mg/kg of ketamine/1.0 mg/kg of ketamine	0.5 mg/kg of ketamine/1.0 mg/kg of saline	General anesthesia	After induction	Major open cardiac Or non-cardiac surgeries	70 ± 7.2/70 ± 7.2/70 ± 6.9	143:83/139:84/134:87	—	—
Li	2019	40/40	0.5 mg/kg of ketamine	0.5 mg/kg of saline	General anesthesia	After induction	Breast cancer	46.7 ± 11.0/45.2 ± 10.3	0:40/0:40	121.2 ± 21.4/128.0 ± 23.9	89.2 ± 17.2/95.0 ± 16.4
Peirong	2020	102/100	0.125 mg/kg of ketamine	0.125 mg/kg of saline	General anesthesia	After induction	Breast cancer	47.7 ± 9.7/48.0 ± 10.2	0:102/0:100	137.3 ± 17.6/134.7 ± 16.1	405.8 ± 100/420.6 ± 100.4
Wang	2020	30/30	0.3 mg/kg of ketamine	0.3 mg/kg of saline	General anesthesia	After induction	Rectal cancer	48.39 ± 5.96/48.42 ± 5.95	18:12/19:11	175.48 ± 31.59/189.72 ± 33.51	—

**TABLE 3:** Depression scores of each article included in this meta-analysis.

Author	Hamilton depression rating score	Edinburgh postnatal depression scale	Postpartum depression screening scale	Patient health questionnaire-9	Depressive patients	Visual analogue scale score	Brain-derived neurotrophic factor (ng/ml)	Complication
Akira	9.9 ± 4.1/14.4 ± 3.8/4.8 ± 1.6 (1 day)	—	—	—	—	—	—	—
Liu	—	6.0 ± 3.7/4.3 ± 3.1 (3 days)	—	—	8/2	—	—	5/3
Lv	—	4.0 ± 2.8/6.4 ± 2.1 (3 days)	—	—	2/9	—	—	3/5
Zhang	—	—	57.07 ± 13.97/71.57 ± 30.49 (4 days)	—	2/9	—	—	—
Min	—	—	—	2.68 + 0.73/3.37 + 0.4 (1 day); 2.28 + 0.61/2.92 + 0.63 (5 days)	—	2.17 ± 0.24/3.75 ± 0.42 (1 day); 1.12 ± 0.87/1.88 ± 0.01 (5 days)	26.85 ± 0.74/25.31 ± 0.51 (1 day); 27.12 ± 0.60/25.67 ± 0.74 (5 days)	8/8
Zhou	7.67 ± 2.53/10.48 ± 2.48 (5 days); 7.96 ± 2.61/11.24 ± 2.74 (10 days)	—	52.58 ± 13.36/71.34 ± 15.19 (5 days); 53.73 ± 13.28/73.41 ± 15.37 (10 days)	—	3/11	—	—	2/10
Dai	—	—	—	1.7 ± 1.6/3.2 ± 2.0 (5 days)	2/9	—	—	—
Xu	12.25 ± 4.50/18.64 ± 3.83 (1 day); 10.64 ± 4.33/16.27 ± 4.45 (3 days); 13.45 ± 5.21/17.36 ± 6.25 (7 days)	—	—	—	—	—	—	14/17
Yang	—	7.2 ± 3.9/7.2 ± 4.2 (3 days); 5.6 ± 3.9/5.7 ± 4.3 (6 weeks)	—	—	41/46 (3 days); 26/29 (6 weeks)	—	—	33/14
Mashour	—	—	—	—	24/22/18	—	—	—
Li	—	—	—	—	17/18 (1 day); 15/22 (3 days)	—	—	—
Peirong	13.2 ± 2.50/16.4 ± 2.0 (3 days); 10.5 ± 2.9/11.2 ± 3.6 (7 days); 9.5 ± 2.9/11.0 ± 3.8 (1 month); 7.5 ± 3.2/7.5 ± 3.0 (3 months)	—	—	—	—	3.4 ± 0.7/4.0 ± 0.8 (1 day); 2.7 ± 0.6/3.2 ± 0.7 (3 days); 1.3 ± 0.2/1.3 ± 0.2 (7 days)	23.1 ± 1.2/21.6 ± 1.5 (3 days); 23.5 ± 1.2/21.5 ± 1.6 (7 days); 24.1 ± 1.4/23.0 ± 1.2 (1 month); 27.1 ± 1.5/26.9 ± 1.5 (3 months)	37/40
Wang	—	—	47.44 ± 5.06/40.51 ± 4.46 (1 day)	—	—	2.5 ± 0.55/4.78 ± 1.34 (1 day)	—	0/5

### Meta-Analysis

#### Anesthesia Duration

**FIGURE 2:**
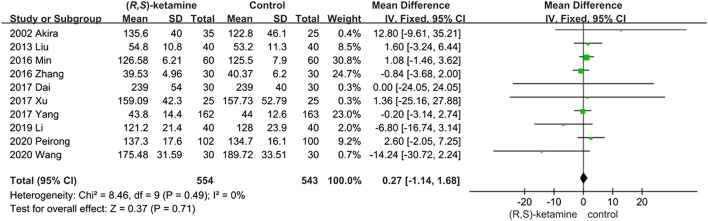
Anesthesia duration forest plot.

Ten studies reported the anesthesia duration of (*R*,*S*)-ketamine and control interventions. A total of 554 and 543 patients were treated with a small dose of (*R*,*S*)-ketamine or an equal volume of saline, respectively. Anesthesia duration did not significantly differ between groups (OR: 0.27; 95% CI: −1.14 to 1.68; P = 0.71; Figure 2).

#### Blood Loss

**FIGURE 3:**
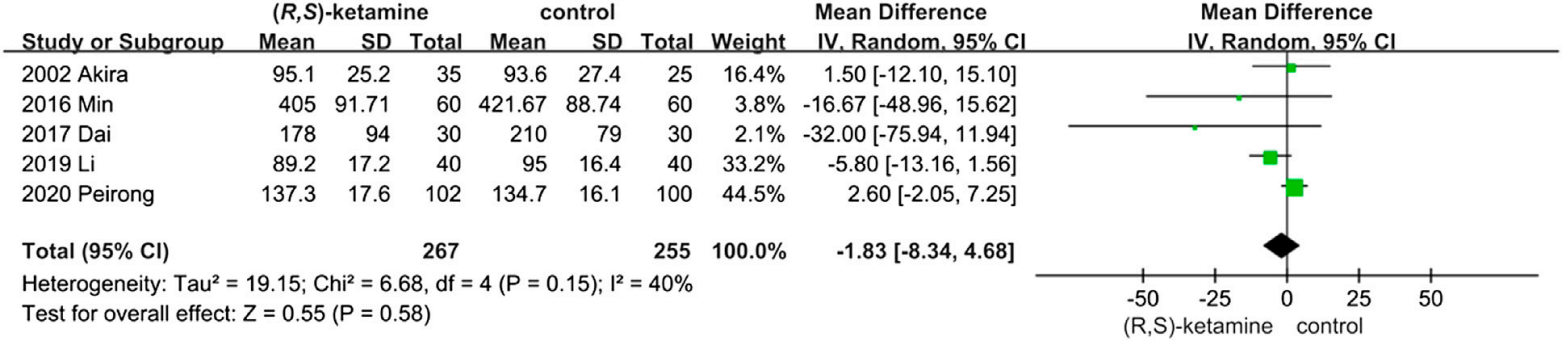
Blood loss forest plot.

Five trials included blood loss data to evaluate surgery safety. Blood loss did not differ significantly between the two groups (OR: −1.83; 95% CI: −8.34 to 4.68; P = 0.58; Figure 3), indicating that a small dose of (*R*,*S*)-ketamine did not affect blood loss during surgery.

#### Depressive Patients

**FIGURE 4:**
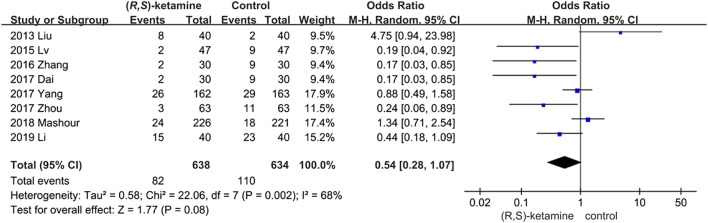
Forest plot of depressive patient data.

We identified eight trials with relevant data. The number of postoperative depressive patients in the (*R*,*S*)-ketamine group (82 of 638) was less than that in the control group (100 of 634; 95% CI: 0.8–1.07; P = 0.08; ketamine: control = 12.9%:15.8%), but the difference was not significant (Figure 4). Sensitivity and subgroup analyses of these results were performed because of the high heterogeneity found in this analysis (*I* ^*2*^ = 68%) (see sensitivity and subgroup analyses part).

#### Postoperative Complications

**FIGURE 5:**
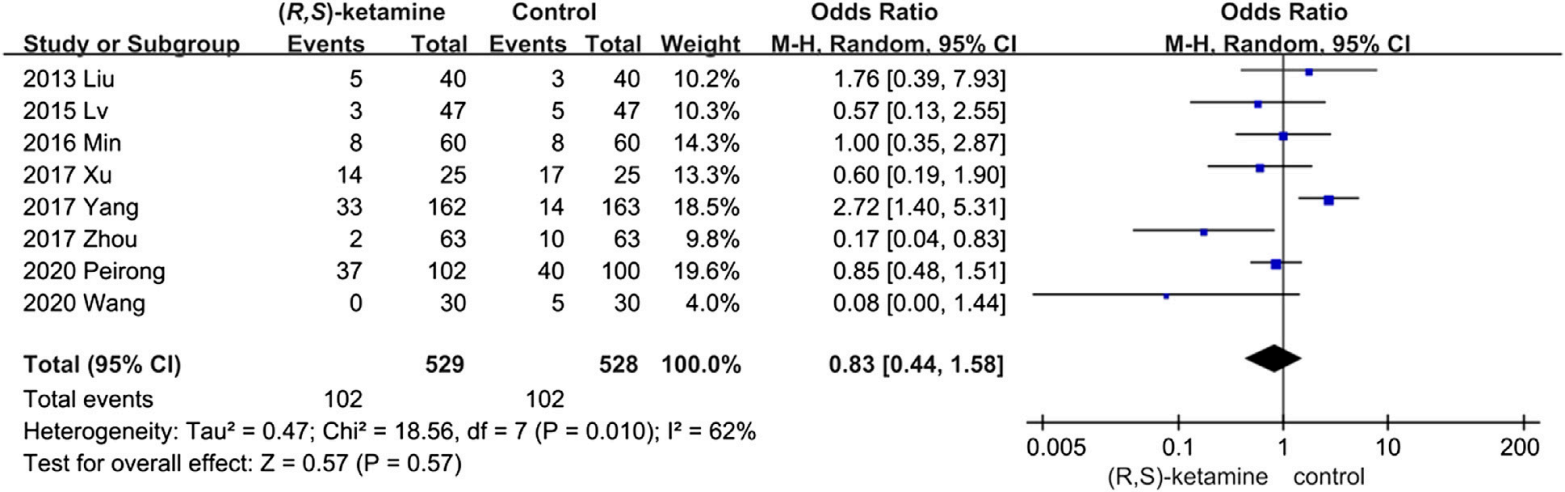
Forest plots of meta-analysis: Postoperative complications.

The incidence of postoperative complications was evaluated in eight studies but did not differ significantly between the (*R*,*S*)-ketamine and control groups (OR: 0.83; 95% CI: 0.44–1.58; P = 0.57; ketamine: control = 19.28%:19.31%; Figure 5). We performed sensitivity and subgroup analyses of the results because of the high heterogeneity. The studies were divided into two subgroups: age and anesthesia mode (*I* ^*2*^ = 62%; see the sensitivity analysis and subgroup analysis subsection). However, the occurrence of postoperative complications did not significantly differ between subgroups.

#### Extubation Time

**FIGURE 6:**
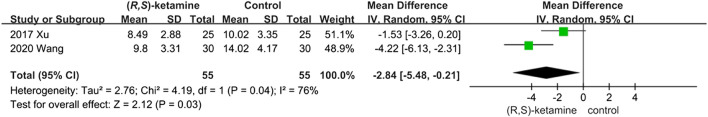
Extubation time forest plot.

Although only two articles discussed this result, we found that intraoperative low-dose (*R*,*S*)-ketamine reduced extubation time (OR: −2.84; 95% CI: −5.48 to −0.21; P = 0.03; Figure 6).

#### Publication Bias

**FIGURE 7:**
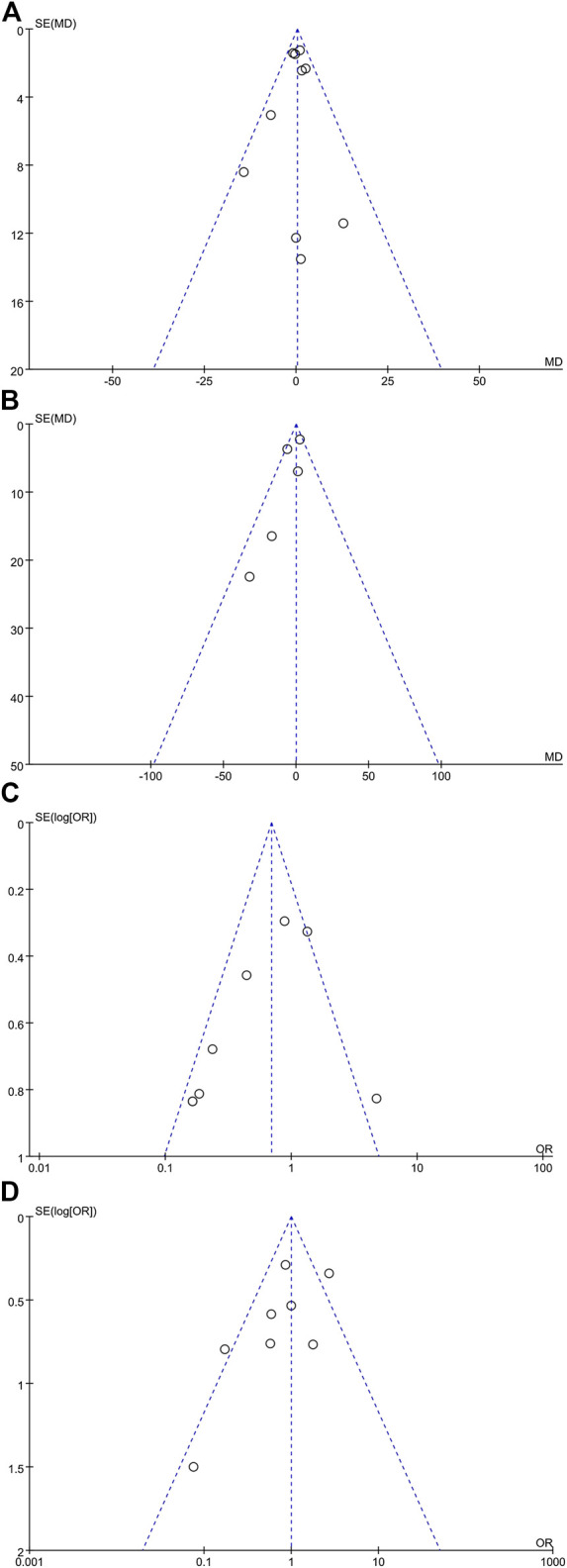
Meta-analysis funnel plots. **(A)** Anesthesia duration meta-analysis (10 articles), **(B)** blood loss meta-analysis (five articles), **(C)** depressive patient meta-analysis (nine articles), and **(D)** postoperative complication meta-analysis (eight articles).

The funnel plot shapes for anesthesia duration, blood loss, depressed patients, and postoperative complications showed basic symmetry. No significant publication bias was observed. The results were similar, and the combined results were highly reliable (Figure 7).

#### Depression Score

**FIGURE 8:**
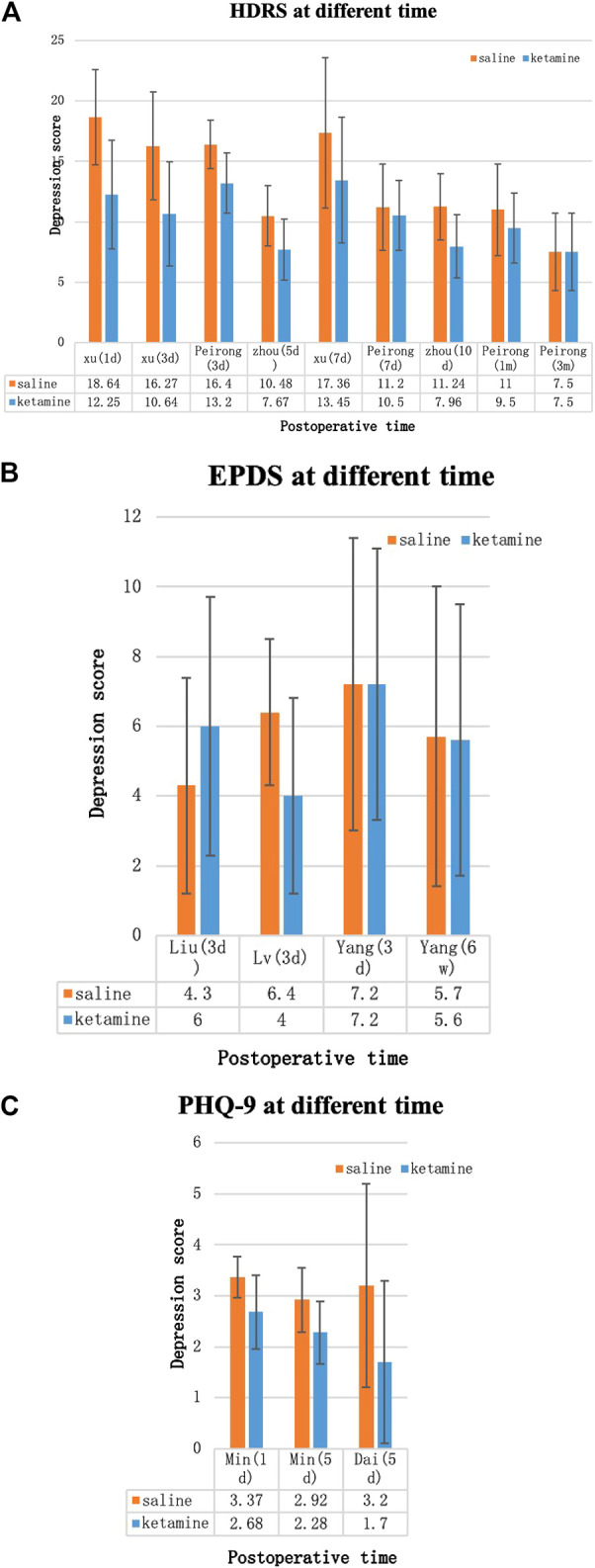
Depression scores over time. **(A)** Hamilton Depression Rating Score (HDRS), **(B)** Edinburgh Postnatal Depression Scale (EPDS), and **(C)** Patient Health Questionnaire-9 (PHQ-9). Blue bars: (*R*,*S*)-ketamine, orange bars: saline. Error bars indicate SD.

**FIGURE 9:**
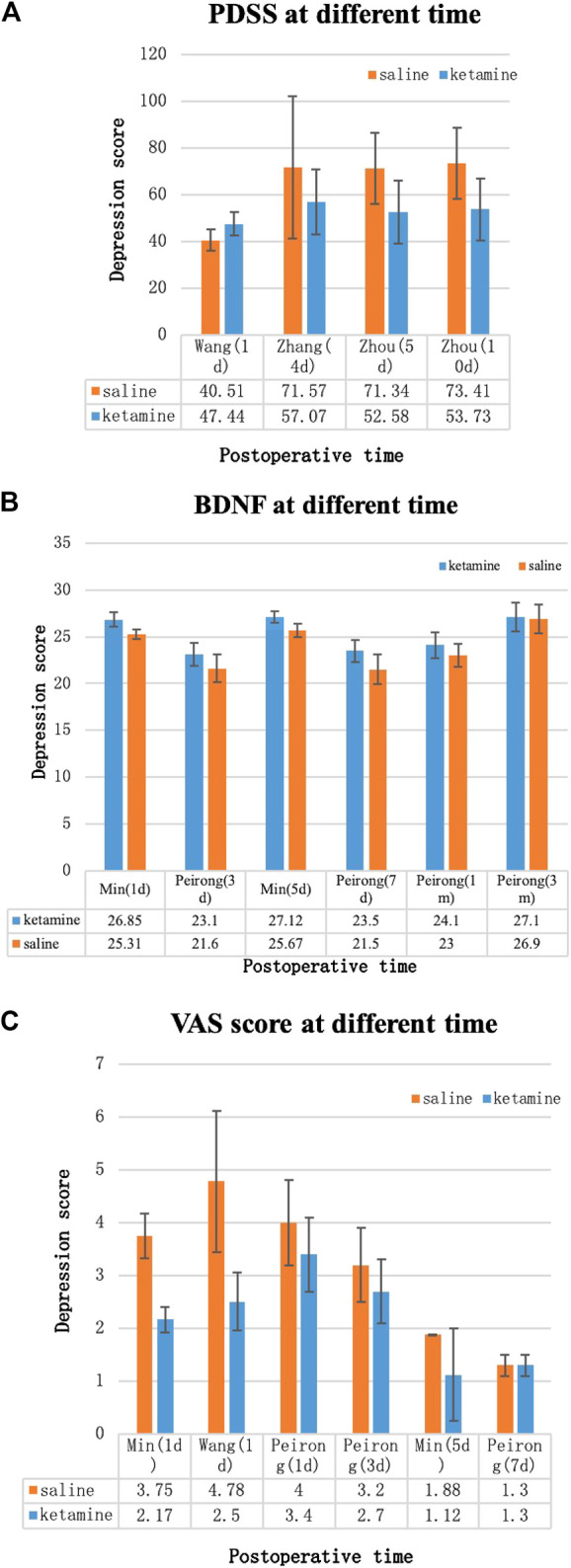
Depression scores over time. **(A)** Postpartum depression screening scale (PDSS), **(B)** brain-derived neurotrophic factor (BDNF) levels, and **(C)** Visual analogue scale (VAS) score. Blue bars: (*R*,*S*)-ketamine, orange bars: saline. Error bars indicate SD.

Because different depression scores were used by different authors, we were unable to perform a meta-analysis on depression scores. Some of the results [Hamilton Depression Rating (HDR) score, Edinburgh Postnatal Depression Scale (EPDS), postpartum depression screening scale (PDSS), Patient Health Questionnaire-9, VAS score, and BDNF] were collected into histograms to identify differing trends between (*R*,*S*)-ketamine and saline treatments (Figures 8, 9). Compared to the control, (*R*,*S*)-ketamine improved HDRS within 1 month in several studies (Figure 7A). However, studies by Liu et al. and Lv et al. showed contradictory results regarding the effect of (*R*,*S*)-ketamine on EPDS (Figure 7B). Liu et al. reported that (*R*,*S*)-ketamine increased EPDS, while Lv et al. reported that (*R*,*S*)-ketamine reduced EPDS. In contrast, Yang et al. suggested that (*R*,*S*)-ketamine did not affect EPDS on day 3 and day 6. Studies by Min and Dai reported that (*R*,*S*)-ketamine reduced Patient Health Questionnaire-9 on day 1 and day 5 (Figure 7C). Wang et al. reported that (*R*,*S*)-ketamine increased PDSS on day 1, while studies by Zhang and Zhou reported that (*R*,*S*)-ketamine decreased PDSS on days 4, 5, and 10 (Figures 8A,B). Min and Peirong reported that (*R*,*S*)-ketamine increased the BDNF level from day 1 postoperation and sustained the increase for 3 months (Figure 8C). Studies by Min, Wang, and Peirong suggested that *S*-ketamine decreased VAS on days 1–5 but that this action weakened and was not significant from day 7 onward.

### Sensitivity Analysis and Subgroup Analysis

**FIGURE 10:**
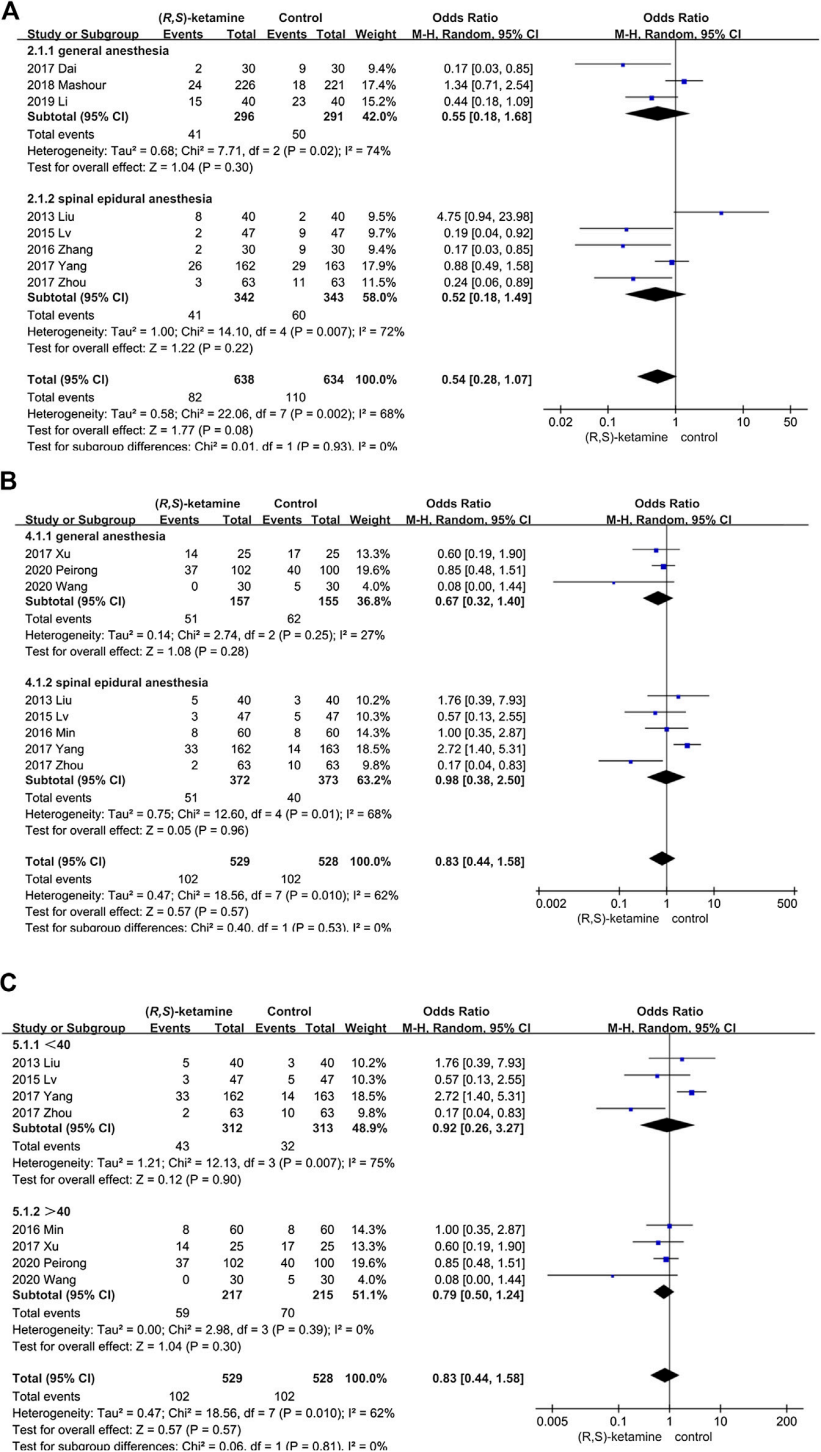
Forest plots for sensitivity and subgroup analyses. **(A)** Analysis of depressive patients according to anesthesia mode. **(B)** Subgroup of postoperative complications according to anesthesia mode. **(C)** Subgroup of postoperative complications according to patient age.

Sensitivity and subgroup analyses were performed for depressive patients and postoperative complications by evaluating differences in the outcome and significance using fixed and random effects models for the meta-analysis. The studies were divided into two subgroups: age (older or younger than 40 years) and anesthesia method (general anesthesia or spinal anesthesia). The statistical significance of these subgroups did not differ in depressed patients, suggesting that the different depression rating methods might have been responsible for this result. In the postoperative complications group, patients who were younger than 40 years or underwent spinal anesthesia were more susceptible to postoperative complications such as dizziness and nausea (P = 0.007 and 95% CI: 0.26–3.27 in the less than 40-year group; P = 0.01 and 95% CI: 0.38–2.50 in the spinal anesthesia group (Figure 10).

## Discussion

The stress of surgery may affect physiological function, and poor postoperative recovery may similarly result in altered physical function. New depressive symptoms may occur after surgery in healthy individuals, and symptoms may worsen in previously depressed patients. Surgery is generally accompanied by the use of various perioperative drugs. This meta-analysis included the literature that explored the prophylactic effect of intravenous low-dose (*R*,*S*)-ketamine (0.5 mg/kg) for postoperative depression based on the theory that low-dose (*R*,*S*)-ketamine is associated with rapid relief of depressive symptoms (Murrough, 2012) and the elimination of suicidal ideation (Bartoli et al., 2017). Some researchers chose to give small doses of (*R*,*S*)-ketamine or *S*-ketamine to patients undergoing general anesthesia and spinal anesthesia and evaluated depression scores at different time points after surgery to determine whether (*R*,*S*)-ketamine effectively prevents postoperative depression.

In general, conclusions differed among studies. Some studies evaluating general anesthesia showed that intravenous (*R*,*S*)-ketamine prevents postoperative depression. Lee reported that (*R*,*S*)-ketamine (0.5 mg/kg) administered after induction relieves postoperative depression in laparoscopic gynecological surgery (Lee et al., 2018). Similarly, Jiang et al. found that 0.5 mg/kg of (*R*,*S*)-ketamine during surgery reduces postoperative depression scores and elevates serum BDNF levels, indicating that (*R*,*S*)-ketamine may alleviate postoperative depression by increasing BDNF expression (Li et al., 2019). Wang et al. found that a single dose of (*R*,*S*)-ketamine (0.4 mg/kg) administered immediately after surgery reduces the SF-MPQ score (Wang et al., 2019).

In contrast, Mashour et al. evaluated the response of individuals older than 60 years who underwent major surgery and found that surgery is associated with the novel onset of postoperative depression in individuals older than 60 years and that 0.5 mg/kg of (*R*,*S*)-ketamine does not prevent or improve depressive symptoms (Mashour et al., 2018). In consistency with Mashour et al.’s work, in an Ochs-Ross et al.’s randomized double-blind study, subjects of ≥65 years with treatment-resistant depression without psychosis were given a flexibly dosed esketamine nasal spray. The primary outcome measure, a change in Montgomery-Asberg Depression Rating Scale scores from baseline to day 28, did not reach statistical significance, and subgroup analysis based on age showed poorer improvement in ≥75 age-group than younger age-groups (Ochs-Ross et al., 2020), indicating S-ketamine may not be an effective antidepressant in elderly population. One possible reason is that the rate of response for antidepressants in older patients decreases with age (Tedeschini et al., 2011). Moreover, antidepressants are found to be ineffective in depression related to dementia (Nelson and Devanand, 2011). One explanation could be white matter hyperintensities in elderly people may be associated with poorer response to antidepressant treatment (Respino et al., 2019). These factors make the prevention of postoperative depression increasingly difficult. Ma et al. and Zhou et al. reported that 0.5 mg/kg of (*R*,*S*)-ketamine can reduce postpartum depression after cesarean section (Zhou and Gan, 2017; Ma et al., 2019), while Lv and Liu et al. reported that 0.25 mg/kg of (*R*,*S*)-ketamine can prevent postpartum depression after cesarean delivery (Liu and Zhang, 2013; L, 2015). However, Xu et al. found that 0.25 mg/kg of (*R*,*S*)-ketamine did not prevent postpartum depression (Xu Y. et al., 2017). It is possible that the concentration used by Xu et al. was too small to exert an antidepressant effect.

The meta-analysis results conclusively suggest that low-dose (*R*,*S*)-ketamine does not change depression scores after cesarean section with spinal anesthesia. Instead, it increases the incidence of adverse reactions, such as dizziness and drowsiness, in patients younger than 40 years, which suggests that patients younger than 40 years may be more sensitive to the side effects of (*R*,*S*)-ketamine; this may be related to the fact that younger individuals have higher N-methyl-D–aspartate receptor signaling pathway activity that changes with age (Xiao et al., 2011). However, there was a trend that indicated intra-operative (*R*,*S*)-ketamine administration could prevent postoperative depression, although it was not statistically significant (P < 0.08). It is noteworthy that despite the failure to meet significance on the outcome, there were decreases in the number of patients showing depressive symptoms in the ketamine group. One reason for failure to meet significance could be the small sample size and relatively lower dosage in some of the included trials. We hypothesize that increasing the dose of (*R*,*S*)-ketamine may produce antidepressant effects in ketamine-ineffective populations, but it may also increase the occurrence of adverse events. Another purported reason is that the inclusion of the elderly patients who are relatively resistant to antidepressant such as ketamine, as late-onset depression is associated with greater treatment resistance (Naismith et al., 2012). Although the results failed to meet significance on primary outcome measure, it still showed possible benefit for ketamine in reducing depressive symptoms.

One reason (*R*,*S*)-ketamine may not have significantly prevented postoperative depression is potential interactions with other anesthetic drugs. Physiological status is significantly changed during general anesthesia. Administering (*R*,*S*)-ketamine during general anesthesia and administering (*R*,*S*)-ketamine alone produce differing effects in the cortex. This may be the reason for the differing antidepressant effects of (*R*,*S*)-ketamine in general anesthesia compared to those in spinal anesthesia. For instance, administering (*R*,*S*)-ketamine alone increases high-frequency cortical oscillations at a low dose (Pal et al., 2015). However, when low-dose (*R*,*S*)-ketamine is given along with general anesthesia, high-frequency cortical power remains inhibited (Hambrecht-Wiedbusch et al., 2017). Furthermore, although (*R*,*S*)-ketamine inhibits burst firing in the lateral habenula, which may account for its rapid antidepressant effect (Yang et al., 2018), recent studies suggest that many anesthetics can activate lateral habenula glutamatergic neurons (Mashour and Kelz, 2018). Thus, (*R*,*S*)-ketamine may not exert a satisfactory antidepressant effect under general anesthesia conditions. Studies have shown that propofol can reduce astrocyte secretion of BDNF (Liu et al., 2017), while dexmedetomidine can activate the Erk1/2/CREB/BDNF signal transduction pathway (Tu et al., 2019). We postulate that the combination of (*R*,*S*)-ketamine and dexmedetomidine may enhance the antidepressant effect. Sevoflurane can exert antidepressant effects by blocking the HMGB1/TLR4 signal transduction pathway (Guo et al., 2019). Therefore, the combined use of sevoflurane and (*R*,*S*)-ketamine during surgery may enhance the antidepressant effect. Halothane and diazepam can delay the biotransformation of (*R*,*S*)-ketamine and prolong its action time, while opioid antagonists can attenuate the effects of (*R*,*S*)-ketamine (Williams et al., 2019). Postoperative multimodal analgesia may also cover the antidepressant effect of (*R*,*S*)-ketamine since it alleviates the analgesic action of (*R*,*S*)-ketamine. In addition to the mechanisms of surgical stress and the effects of narcotic drugs, it is possible that postpartum depression caused by sex hormone fluctuations also participates in the postoperative depression mechanism (Gordon et al., 2015).

*S-*Ketamine and *R-*ketamine exert different antidepressant effects. *S-*Ketamine precipitates behavioral abnormalities such as hyperlocomotion, while *R-*ketamine exerts sustained antidepressant effects through BDNF-TrkB signaling and synaptogenesis in the PFC and CA3 regions. Compared to *S*-ketamine, *R-*ketamine appears to be a safer and more potent antidepressant drug (Yang et al., 2015). Of the 13 studies included in this meta-analysis, only one (Peirong) reported the type of ketamine used (*S*-ketamine). It is possible that the type of ketamine could have affected the results of the analysis.

Variations in patient baseline conditions may also have contributed to the differing results. The antidepressant effect of (*R*,*S*)-ketamine is affected by baseline inflammation levels, metabolic pathway activation levels (Ionescu et al., 2014; Zhou et al., 2020), immune status (Zhang et al., 2020), baseline depression levels (Pal et al., 2015; Stoker et al., 2019), baseline functional connectivity of the brain (Li et al., 2020), family drinking history (Pennybaker et al., 2017), gender (Picard et al., 2019), and age. Moreover, (*R*,*S*)-ketamine may reduce depression scores by alleviating pain (Riddell et al., 2019; Thompson et al., 2019). However, this effect may not be significant in patients with severe pain.

In addition to the effects of (*R*,*S*)-ketamine on depression, studies have also covered some of the side effects of (*R*,*S*)-ketamine. (*R*,*S*)-ketamine causes intra-operative hemodynamic changes; it increases systolic and diastolic blood pressure, which returns to normal levels 2–4 h after administration (Szarmach et al., 2019). This suggests that (*R*,*S*)-ketamine may increase intra-operative bleeding. However, the results of the current study suggested that (*R*,*S*)-ketamine did not affect intra-operative blood loss. This is consistent with the conclusions of Xiang et al., who suggested that (*R*,*S*)-ketamine does not affect compensatory vasoconstriction after bleeding (Xiang et al., 2020). The uncertain effect of (*R*,*S*)-ketamine on surgical bleeding may also be related to the combined use of other narcotic sedative drugs such as barbiturates and benzodiazepines. The results of our meta-analysis suggested that (*R*,*S*)-ketamine reduced extubation time. Abdolahi reported that (*R*,*S*)-ketamine can shorten extubation time (Abdolahi et al., 2013), but the specific mechanism for this is unclear. Mildh et al. (1998) reported that 0.25 mg/kg (*R*,*S*)-ketamine can reverse the decline in ventilation caused by fentanyl (76), suggesting that (*R*,*S*)-ketamine may accelerate spontaneous ventilation recovery to reduce extubation time. In animal experiments, (*R*,*S*)-ketamine can also shorten the recovery time of isoflurane-anesthetized rats (Hambrecht-Wiedbusch et al., 2017). The side effects of (*R*,*S*)-ketamine include feelings of strangeness, dizziness, diplopia, nystagmus, cognitive impairment, urinary tract symptoms, and addictive side effects. Most side effects peaked at 1 h after injection and most resolved at 2 h after injection (Acevedo-Diaz et al., 2020). Other side effects include central nervous system excitement, sedation, and visual disturbance (Schwenk et al., 2016). The mechanism of these side effects remains elusive and may be related to the increased cerebral blood flow and increased intracranial pressure caused by (*R*,*S*)-ketamine. Symptoms such as dizziness and nausea caused by spinal anesthesia may cause patients to be more sensitive to side effects of (*R*,*S*)-ketamine.

### Limitations

This review provides a comprehensive comparison of intraoperative low-dose (*R*,*S*)-ketamine administration and its effects. This is one of the few meta-analyses to explore whether (*R*,*S*)-ketamine can prevent depressive symptoms after surgery. However, limitations of the current study include the following: first, publication and selection bias could be a substantial issue. The included studies were published either in Chinese or English, and most described positive results, which could have led to publication bias. Variation in patient selection and the methods of assessing complications could have caused a selection bias. Second, the small number of patients and studies decreases the reliability of the findings, even though several databases were searched. A greater number of studies should be evaluated, and the results must provide more effective evidence in high-quality trials. Furthermore, the use of different depression-rating scales may have influenced the number of postoperative patients. Finally, we did not discuss the prognosis and long-term complications of (*R*,*S*)-ketamine administration.

## Conclusion

(*R*,*S*)-Ketamine has no prophylactic effect on postoperative depression in patients undergoing general anesthesia or spinal anesthesia, but it does lead to a higher incidence of adverse reactions in young patients (less than 40 years) undergoing cesarean section under spinal anesthesia. These results suggest that low-dose intravenous (*R*,*S*)-ketamine during surgery may be of little significance in preventing postoperative depression but may increase postoperative discomfort in young female patients. The prophylactic effects of (*R*,*S*)-ketamine on postoperative depression need to be further explored and should evaluate the optimal administration route (intravenous, intramuscular, oral, or nasal spray), the dosage, and the timing (preoperative, intraoperative, and immediate postoperative) as well as the drug resistance and sensitivity of different patients as these factors may have a significant effect.

## Author Contributions

LP: literature research, manuscript preparation; MC: literature research, manuscript preparation; WD: literature research; JK: literature research; HC: manuscript final version approval; SW: manuscript final version approval.

## Funding

This study was funded by the National Nature Science of China (81670580) and 345 Talent Project.

## Conflict of Interest

The authors declare that the research was conducted in the absence of any commercial or financial relationships that could be construed as a potential conflict of interest.
